# Long non-coding RNA BZRAP1-AS1 silencing suppresses tumor angiogenesis in hepatocellular carcinoma by mediating THBS1 methylation

**DOI:** 10.1186/s12967-019-02145-6

**Published:** 2019-12-17

**Authors:** Weiwei Wang, Guoyong Chen, Bing Wang, Zhenhua Yuan, Guangbo Liu, Biao Niu, Yongfeng Chen, Shaotang Zhou, Junchuang He, Huanzhou Xue

**Affiliations:** Department of Hepatobiliary Surgery, People’s Hospital of Zhengzhou University, Henan Provincial People’s Hospital, No. 7, Weiwu Road, Jinshui District, Zhengzhou, 450003 People’s Republic of China

**Keywords:** Hepatocellular carcinoma, Long non-coding RNA, BZRAP1-AS1, DNA methyltransferase 3B, Thrombospondin-1, DNA methylation, Angiogenesis

## Abstract

**Background:**

Hepatocellular carcinoma (HCC) is the most frequent primary liver cancer associated with a high mortality. Long non-coding RNAs (lncRNAs) have recently emerged as regulators in the development and progression of several cancers, and therefore represent an opportunity to uncover new targets for therapy. In the present study, we aimed to investigate the potential effect of lncRNA BZRAP1-AS1 on the angiogenesis of HCC.

**Methods:**

Microarray-based data analysis was initially employed to screen genes and lncRNAs that are differentially expressed in HCC and the candidate BZRAP1-AS1 was identified as a hit. The expression of BZRAP1-AS1 and thrombospondin-1 (THBS1) in HCC tissues and cells were then determined using RT-qPCR. The gene methylation level was measured by methylation-specific PCR (MSP) and bisulfite sequencing PCR (BSP) assays. Next, the interactions between BZRAP1-AS1, DNA methyltransferase 3B (DNMT3b), and THBS1 were assessed by RIP, RNA pull-down and ChIP assays. Finally, the roles of BZRAP1-AS1, DNMT3b and THBS1 in angiogenesis in vitro as well as tumorigenesis in vivo were evaluated by a battery of the gain- and loss-of function experiments.

**Results:**

BZRAP1-AS1 was identified as a highly expressed lncRNA in HCC tissues and cells. Down-regulation of BZRAP1-AS1 in HCC cells inhibited HUVEC proliferation, migration and angiogenesis. By interacting with DNMT3b, BZRAP1-AS1 induced methylation of the THBS1 promoter and inhibited the transcription of THBS1, resulting in promoted angiogenesis of HUVECs. Moreover, silencing of BZRAP1-AS1 repressed the angiogenesis as well as the tumor growth of HCC in vivo via up-regulating THBS1.

**Conclusion:**

This study provides evidence that angiogenesis in HCC is hindered by silencing of BZRAP1-AS1. Thus, BZRAP1-AS1 may be a promising marker for the treatment of HCC.

## Background

Liver cancer is one of the most frequent cancers associated with high cancer-specific deaths and an increasing incidence worldwide [1]. Hepatocellular carcinoma (HCC) has been reported to be the most common type of primary liver cancer [2]. There is a disproportionately large incidence of HCC in Asia, mainly attributed to the endemic status of chronic hepatitis B virus (HBV) and hepatitis C virus (HCV) infections [3]. Despite the improvements made in nonsurgical treatment platforms and standardization of diagnosis, the treatment of patients with HCC is confronted with challenges caused by patient-, tumor- and liver-specific variables [4]. Although liver transplantation has been a widely prescribed therapy for patients with early-stage HCC, the guidelines are still controvertible [5]. Genetic analyses of liver tissues can potentially be beneficial to individualized management of HCC and improved classification of HCCs according to their molecular features [6]. Aside from microRNAs (miRNAs or miRs) which are the most widely studied non-coding RNAs (ncRNAs) thus far, long non-coding RNAs (lncRNAs) are increasingly gaining recognition as important regulators of gene transcription [7]. A better understanding of lncRNAs in the progression of HCC therefore presents an opportunity for the identification of novel therapeutic targets for its diagnosis and treatment.

LncRNAs, an extensive set of structurally complex RNAs, can mediate gene expression through interacting with DNA, RNA, or protein molecules and exert cellular functions in HCC via diverse mechanisms [8]. A recent study has identified a number of deregulated lncRNAs, such as TEX41, HAND2-AS1 and XLOC_014515, some of which have been experimentally validated and mechanistically associated with cancer development and progression [9]. Another energy stress-inducible lncRNA MITA1 is a metastatic lncRNA in HCC [10]. A novel lncRNA, benzodiazapine receptor associated protein 1 antisense RNA 1 (BZRAP1-AS1) has been previously identified as a biomarker associated with prostate cancer [11]. Thrombospondin family contains five secreted proteins which play diverse modulatory roles in cellular function [12]. As a member of this family, thrombospondin-1 (THBS1) (also known as TSP1) is the first identified endogenous antiangiogenic factor that might suppress tumor growth through inhibiting tumor angiogenesis that is a critical process in cancer pathogenesis and treatment [13, 14]. Over-expression of THBS1 in the T-cell compartment inhibits angiogenesis, thereby restraining tumor growth [15]. This study aims to investigate the biological functions of lncRNA BZRAP1-AS1 in tumor angiogenesis in HCC and the underlying mechanisms. Our results mainly revealed that BZRAP1-AS1 was an up-regulated lncRNA in HCC, and its expression was negatively correlated with the anti-angiogenic gene THBS1. BZRAP1-AS1 negatively regulated THBS1 through inducing methylation, thus promoting tumor angiogenesis.

## Methods and materials

### Ethics statement

The study protocol was approved by the Ethics Committee and Experimental Animal Ethics of People’s Hospital of Zhengzhou University, Henan Provincial People’s Hospital. Informed written consent was obtained from each participant prior to the study. The animal experiment strictly adhered to the principle to minimize the pain, suffering and discomfort to experimental animals.

### Microarray-based gene expression analysis

The datasets of HCC-related microarrays (GSE33006, GSE45267, GSE49515 and GSE89377) were retrieved from the Gene Expression Omnibus database (https://www.ncbi.nlm.nih.gov/geo/) and annotated in GPL570-[HG-U133_Plus_2] Affymetrix Human Genome U133 Plus 2.0 Array platform. GSE33006 dataset included 3 normal samples and 3 tumor samples, GSE45267 dataset included 17 normal samples and 15 tumor samples, GSE49515 dataset included 10 normal samples and 10 tumor samples, and GSE89377 dataset included 13 normal samples and 35 tumor samples. Raw data were pretreated using the R programming language available at http://www.r-project.org, and normalized gene expression was obtained using the RMA algorithm of the Bioconductor Affy package [16]. Thereafter, the differential analysis of gene expression was performed using the “Limma” package of R language. Then the screening of differentially expressed genes related to HCC was carried out with |log2 fold change| > 1 and *p* value < 0.05 set as the threshold. Next, a heatmap of the differentially expressed genes was plotted by means of “pheatmap” package of R language.

### Patient enrollment

A total of 49 patients (36 males and 13 females; mean age of 55.12 ± 10.91 years) were pathologically diagnosed as primary HCC and underwent surgical resection at the People’s Hospital of Zhengzhou University (Henan Provincial People’s Hospital) from January 2015 to December 2017. In addition, the adjacent normal tissues were collected from 20 cases of HCC patients as controls (separated from ≥ 2 cm from the tumor margin and were confirmed without tumor cells under a microscope). None of patients received anticancer treatment before surgery. The tumor nodules were completely resected. Complete clinical and follow-up data were collected for all patients. Patients were excluded in this study if they died of non-liver diseases or accidents. The differentiation of cancer cells was histologically graded according to the Edmondson-Steiner grading: HCC grade I–II was observed in 32 cases and HCC grade III–IV was observed in 17 cases. Moreover, based on the tumor-node-metastasis staging, 27 cases were at the clinical stage I, 12 cases at the clinical stage II, and 10 cases at the clinical stage III.

### Cell culture and treatment

Human normal liver cells L-02 and human HCC cell lines (HuH-7, HCCLM3, LI7, BEL-7405, SK-HEP-1 and BCLC-9) were purchased from Shanghai Institute for Biological Sciences, Chinese Academy of Sciences (Shanghai, China) (http://www.cellbank.org.cn/index.asp). SK-HEP-1 cells were cultured in minimum essential medium containing 10% fetal bovine serum (FBS) at 37 °C with 5% CO_2_. HuH-7 and HCCLM3 cells were cultured in Dulbecco’s modified Eagle’s medium (DMEM) containing 10% FBS at 37 °C with 5% CO_2_, and L-02, LI7, BEL-7402 and BCLC-9 cells were cultured in Roswell Park Memorial Institute (RPMI) 1640 medium containing 10% FBS at 37 °C with 5% CO_2_. All medium above were purchased from Gibco BRL (Gaithers burg, MD, USA).

The methyltransferase inhibitor, 5-aza-2′-deoxycytidine (5-aza-dc; Zymo research, Irvine, CA, USA) was used to inhibit DNA methylation at a final concentration of 0.5 μmol/L 5-aza-dc, and 0.5% dimethyl sulfoxide (DMSO) was used as control.

Based on the lentiviral vector pLV-EGFP-N, over-expression lentiviral particles including pLV-EGFP-BZRAP1-AS1 (overexpressed [oe]-BZRAP1-AS1), and pLV-EGFP-THBS1 (oe-THBS1) were constructed. Puromycin was applied to screen the infected cells for stable expression. The short hairpin RNA (shRNA) against BZRAP1-AS1 or DNMT3b was inserted into pSIH1-H1-copGFP vector. The lentiviral vectors including pSIH1-H1-copGFP-sh-BZRAP1-AS1 (sh-BZRAP1-AS1), pSIH1-H1-copGFP-sh-DNMT3b (sh-DNMT3b), and negative control shRNA pSIH1-H1-copGFP-sh-NC (sh-NC) were constructed. The plasmids were constructed by Shanghai GenePharma Co., Ltd. (Shanghai, China). For lentivirus packaging, 293T cells were cultured in complete RPMI 1640 containing 10% FBS and passaged every 2 days. The HCC cells at the logarithmic growth phase were detached with trypsin and dispersed into cell suspension at a density of 5 × 10^4^ cells/mL. Then the cell suspension was inoculated into a 6-well plate (2 mL/well) and cultured overnight at 37 °C. Finally, the cells were infected with the constructed lentiviruses (1 × 10^8^ TU/mL). The infection efficiency was estimated by measurement of the expression of green fluorescent protein (GFP) under a fluorescence microscope 48 h later.

### Reverse transcription quantitative polymerase chain reaction (RT-qPCR)

The total RNA was extracted from HCC cells and tissue samples (30 mg) by Trizol (Sigma-Aldrich Chemical Company, St Louis, MO, USA). UV–visible spectrophotometry was used to determine the quality and concentration of RNA. Then, the extracted RNA was reversely transcribed into complementary DNA (cDNA) using the PrimeScript™ RT Reagent Kit (Takara Bio Inc., Otsu, Shiga, Japan). Subsequently, the quantitative PCR was performed according to the instructions of the SYBR^®^ Premix Ex Taq™ II (Tli RNaseH Plus) kit (Takara Bio Inc., Otsu, Shiga, Japan) in the Thermal Cycler Dice Real Time System (TP800, Takara Bio Inc., Otsu, Shiga, Japan). Primer sequences are shown in Table 1, which were designed using Primer Express 2.0 software and synthesized by Guangzhou RiboBio Co., Ltd. (Guangdong, China). The relative expression was calculated using the 2^−ΔCT^ method with glyceraldehyde-3-phosphate dehydrogenase (GAPDH) as the internal reference.

**Table 1:** Primer sequences for RT-qPCR

Genes	Primer sequences
BZRAP1-AS1	F: 5′-CTGAATCTCGCTTGATCGT-3′
	R: 5′-GAGTAGTCGGAAAGGATGC-3′
GAPDH	F: 5′-CCTGGCCAAGGTCATCCATG-3′
	R: 5′-GGAAGGCCATGCCAGTGAGC-3′
THBS1	F: 5′-GCTCCAGTCCTACCAGTGTC-3′
	R: 5′-TCAGTCACTTGCGGATGCT-3′
DNMT3b	F: 5′-CCAGCTGAAGCCCATGTT-3′
	R: 5′-ATTTGTCTTGAACGCTTG-3′

*F* forward, *R* reverse, *RT-qPCR* reverse transcription quantitative polymerase chain reaction, *GAPDH* glyceraldehyde-3-phosphate dehydrogenase, *THBS1* thrombospondin1, *DNMT3B* DNA methyltransferase 3B

### Western blot analysis

The collected cells were washed with phosphate buffered saline (PBS) and incubated with protein lysis buffer containing protease and alkaline phosphatase inhibitor for 30 min at 4 °C. Next, the cell lysate was obtained. Afterwards, the protein was mixed with the loading buffer, boiled for 5 min and then subjected to 10% sodium dodecyl sulfate-polyacrylamide gel electrophoresis. After that, the proteins were transferred onto a polyvinylidene fluoride membrane. The membrane was then blocked by 5% skim milk powder for 1 h, and incubated overnight at 4 °C with Tris-buffered saline Tween-20 (TBST)-diluted primary rabbit antibody to DNMT3b (ab79822, 1:1000, Abcam Inc., Cambridge, UK). Afterwards, the membrane was incubated with horseradish peroxidase-labeled secondary antibody for 1 h at room temperature. Finally, the membrane was developed by enhanced electrochemiluminescence. β-actin (ab6276, 1:5000, Abcam Inc., Cambridge, UK) served as the internal reference. The gray value of each band was analyzed by the gel image analysis software Image J, and the ratio of the gray value of the target protein to β-actin was calculated.

### Fluorescence in situ hybridization (FISH)

The expression and subcellular location of BZRAP1-AS1 in HCC cells were detected by FISH. In brief, the cells were cultured in a 24-well plate with a density of 5 × 10^3^ cells/well, and the supernatant was removed 24 h later. After being washed with PBS and fixed by 4% paraformaldehyde, the cells were permeabilized with PBS containing 0.5% Triton X-100. Afterwards, the cells were blocked by the pre-hybridization solution at 37 °C, and hybridized overnight with BZRAP1-AS1 specific probe at 37 °C. The cells were washed with the hybridization solution at 42 °C in the dark. Finally, the cells were stained by 4′-6-diamidino-2-phenylindole (DAPI) (Sigma-Aldrich Chemical Company, St Louis, MO, USA). The fluorescence was observed and photographed under a laser confocal microscope.

### Fractionation of nuclear/cytoplasmic RNA

Nuclear and cytoplasm fractions were separated according to the instructions of the PARIS™ Kit Protein and RNA Isolation System (Life Technologies, Carlsbad, CA, USA) kit. The collected HCC cells were washed with PBS and detached with trypsin, and added with 2 mL culture medium to terminate the detachment. The cells were centrifuged at 500*g* for 5 min at 4 °C and the cell pellet was washed with PBS, after which the supernatant was discarded. After the addition of 500 μL of cell fractionation buffer, the precipitation was allowed to stand on ice for 5–10 min and centrifuged at 500*g* for 5 min at 4 °C. The cytoplasmic fractions in the supernatant were transferred into a 2 mL sterile enzyme-free tube and centrifuged at 500*g* for 5 min at 4 °C. The nuclear fractions in pellet were subsequently resuspended in 500 μL of cell fractionation buffer, mixed with 500 μL of 2× lysis/binding solution to discard the supernatant. A total of 500 μL pre-cooled Cell Disruption Buffer and 500 μL of absolute ethanol were then added into the nuclear fractions. Finally, the adsorption column was placed into the collection tube and 700 μL reaction solution was added for centrifugation at 12,000*g* for 30 s for each time with 3 times repeated. The liquid separated at the first and second centrifuge was discarded. The expression of BZRAP1-AS1 was subsequently measured by RT-qPCR with 45S rRNA as the internal reference for nuclear RNA and 12S rRNA for cytoplasmic RNA.

### Cell treatments

HCC cells and human umbilical vein endothelial cells (HUVECs) in logarithmic growth phase were separately treated with a mixture of 0.25% trypsin and 0.02% ethylenediamine tetraacetic acid (EDTA) (1:1). HCC cells and HUVECs were suspended in DMEM respectively to prepare single HCC cell suspension (1 × 10^5^ cells/mL) and single HUVEC cell suspension (5 × 10^5^ cells/mL). HUVECs were then plated to the basolateral chamber at a density of 2 × 10^5^ cells/well and HCC cells were plated to the apical chamber of the 6-well Transwell chamber (0.4 μm pore size; Corning Glass Works, Corning, N.Y., USA). After that, the cells were co-cultured in DMEM containing 10% FBS, at 37 °C with 5% CO_2_ for 48 h. Finally, HUVECs were collected for the subsequent assays.

### 5-Ethynyl-2′-deoxyuridine (EdU) assay

The Cell-Light EdU Imaging Detection Kit (Guangzhou, RiboBio Co., Ltd., Guangdong, China) was used to determine cell proliferation. HUVECs co-cultured with HCC cells were seeded into a 96-well plate at a density of 5 × 10^3^ cells/well. After 6 h, HUVECs were added with EdU-labeled medium and incubated for 60 min after the cells were adhered to the surface of the well. The cells were then fixed with 4% formaldehyde for 15 min and treated with 0.5% Triton X-100 for 20 min. After that, the cells were stained by the Apollo staining reaction solution for 30 min. Finally, the cell DNA was stained with Hoechst 33,342 (5 μL/mL) for 30 min, and images were obtained and the stained cells were quantified under a fluorescence microscope.

### Transwell assay

The in vitro cell migration assay was performed in 24-well plates using Transwell chamber (8 μm pore size; Corning Glass Works, Corning, N.Y., USA). Firstly, the HCC cells suspended in DMEM with 10% FBS were plated into the basolateral chamber at a density of 5 × 10^4^ cells/well and cultured for 24 h. Besides, HUVECs co-cultured with HCC cells were plated into the apical chamber with a density of 3 × 10^4^ cells/well. After incubation for 18 h, the cells in the inner layer of the Transwell microporous membrane were wiped off with a cotton swab and the cells inside the chambers were fixed with methanol, followed by staining with 0.1% crystal violet. The stained cells were counted and photographed under an inverted microscope. A total of 5 visual fields were randomly selected and the average number was calculated.

### Tube formation assay

HUVECs were cultured in EGM-2 medium containing 10% FBS. BD Matrigel™ Matrix was diluted with the serum-free RPMI-1640 medium at a ratio of 1:1. Then, 40 μL of mixture was added onto the upper surface of the porous polycarbonate membrane of Transwell chamber (membrane pore size 8 μm) to form a gel by drying in a fume hood at room temperature for 1 h. Then, HUVEC suspension was added into the apical chamber at a density of 2 × 10^5^ cells/mL and 200 μL/well, and 500 μL of the treated HCC cell supernatant was added to the basolateral chamber. After 24 h of culture in a humidification incubator with 5% CO_2_ at 37 °C, the microvessel-like and tubular branch nodes were counted under an optical microscope with 5 visual fields of view taken from each well.

### Enzyme-linked immunosorbent assay (ELISA)

After culture for 20 h, HCC cells were lysed with 60 μL of cell lysis buffer (Beyotime Biotechnology Co., Shanghai, China). The cell lysate was then sonicated and centrifuged, followed by supernatant collection. The protein concentration was measured according to the instructions of the bicinchoninic acid Protein Concentration Assay Kit (Beyotime Biotechnology Co., Shanghai, China). The optical density (OD) value was measured at a wavelength of 562 nm in the ELISA detector (Vafioskan Flash, Thermo, USA), and the concentration of vascular endothelial growth factor (VEGF) was determined.

### Chicken chorioallantoic membrane (CAM) assay

The chicken embryos were incubated in an electrothermal incubator at 37.8 °C for 7 days, and the well-developed embryo eggs were divided into 8 groups with 15 in each group. An artificial air chamber was made on the surface of the eggshell with a boring machine on a clean bench to expose the urinary capsular membrane tissues rich in blood vessels. The tissues were immediately blocked with sterile tape for subsequent experiments. The HCC cells were detached and counted. Then, 6 × 10^6^ cells in 20 μL of PBS were inoculated into the relatively avascular area of the exposed chicken chorioallantoic membrane, and cultured for another 7 days after being closed with the sterile tape. After that, the chicken embryos were taken out to observe the growth of the embryo blood vessels. CAM transplanted tumor specimens were fixed in 4% paraformaldehyde for 15 min, and the membrane was taken with the transplanted tumor as the center. The membrane was placed on a transparent large glass slide to observe the distribution of neovascularization in the tumor area under a microscope. The blood vessels distributed radially within a range of 5 mm around the tumor tissue were counted.

### Methylation specific PCR (MSP)

The genomic DNA from tissues and cells was extracted using the Genomic DNA purification kit (Qiagen company, Hilden, Germany). Bisulfite modification of DNA was performed by the Intergen CpGenome DNA Modification Kit (Intergen Company, New York, USA) according to the manufacturer’s protocol. THBS1 methylation specific primers were used for MSP (Table 2). The PCR product was then analyzed in a 3% Tris-borate-EDTA agarose gel and the images were processed by the gel imaging system.

**Table 2:** Primer sequences for MSP and BSP

	Primer sequences
THBS1 (M)	F: 5′-TGCGAGCGTTTTTTTAAATGC-3′
	R: 5′-TAAACTCGCAAACCAACTCG-3′
THBS1 (U)	F: 5′-GTTTGGTTGTTGTTTATTGGTTG-3′
	R: 5′-CCTAAACTCACAAACCAACTCA-3′
BSP	F: 5′-GTGGGGTTAGTTTAGGATAGG-3′
	R: 5′-AAAAACACCAAAAAAACCATTC-3′

*THBS1* thrombospondin1, *MSP* methylation specific PCR, *BSP* bisulfite sequencing PCR, *M* methylation, *U* unmethylation, *F* forward, *R* reverse

### Bisulfite sequencing PCR (BSP)

The sulfurized sequencing primers were designed (Table 2). PCR was performed with the same condition as MSP. The PCR product was subjected to agarose gel electrophoresis, and DNA recovery was carried out according to the instructions of agarose gel DNA recovery kit (Tiangen Biotech Co., Ltd, Beijing, China). The DNA fragments were cloned into the pGEM-T-easy (Tiangen Biotech Co., Ltd, Beijing, China). The positive recombinants were selected by PCR using the BcaBEST sequencing Primers and MS universal primers, and then sequenced. The percentage of methylation was expressed as the ratio of the number of methylated cytosine guanine (CG) to the number of detected CG, and averaged.

### RNA binding protein immunoprecipitation (RIP)

When the cell confluence reached 90%, HCC cells (1 × 10^7^) were lysed using the Magna RIP™ RNA-Binding Protein Immunoprecipitation Kit (Millipore, Billerica, MA, USA). Then, the supernatant was collected and divided into 2 equal portions with two copies of Input to detect endogenous proteins in western blot analysis and RNA content in RT-qPCR, respectively. A total of 100 μL cell lysate was incubated overnight at 4 °C for 4 h with 900 μL of RIP buffer containing magnetic beads coated with anti-rabbit primary antibody to DNMT3b (ab2851, 1:1000, Abcam Inc., Cambridge, UK) or immunoglobulin G (IgG). The centrifuge tube was then placed on a magnetic separator to discard the supernatant after centrifugation. After that, the beads were washed with 500 μL of RIP Wash Buffer for 6 times. At the 6th washing, 50 μL was taken out from the 500 μL system to discard the supernatant after which the loading buffer was added and western blot analysis was used to detect if the magnetic beads coated the antibody. The sample was incubated with proteinase K at 55 °C for 30 min and shaken continuously to detach the protein, after which the immunoprecipitated RNA was extracted by Trizol-chloroform to detect the enrichment of BZRAP1-AS1 by RT-qPCR.

### RNA pull-down assay

Biotin-labeled RNA was transcribed by Biotin RNA Labeling Mix (Roche Diagnostics GmbH, Mannheim, Germany) and T7 RNA polymerase (Promega, Madison, WI, USA), treated with RNase-free DNase I (Promega, Madison, WI, USA), and purified by RNeasy Mini Kit (Qiagen company, Hilden, Germany). A total of 3 μg biotinylated RNA was heated to 90 °C for 2 min, cooled on ice for 2 min, and placed at room temperature for 20 min with RNA structure buffer (10 mM Tris pH = 7.0, 0.1 M KCl, 10 mM MgCl_2_). Subsequently, 10^7^ HCC cells were resuspended in 2 mL PBS and incubated on ice for 20 min. The cells were centrifuged at 2500*g* for 15 min with 2 mL of nuclear separation buffer, and 6 mL of H_2_O. Then, the nuclei were resuspended in 1 mL of RIP buffer and sheared by a homogenizer, followed by centrifugation for 10 min and incubation for 1 h at room temperature. Streptavidin agarose beads were added into each binding reaction and incubated with the nuclei for 1 h at room temperature. Finally, the cells were washed 5 times with PBS, added with 5× loading buffer and incubated at 95 °C for 5 min. The DNMT3b protein expression in eluted protein was measured using western blot analysis.

### Dual luciferase reporter gene assay

The promoter sequence of THBS1 as well as the full sequence of lncRNA BZRAP1-AS1 was obtained from the NCBI database (http://www.ncbi.nlm.nih.gov/gene). The sequence of the promoter region of THBS1 amplified gene (− 2000 bp to 50 bp) was cloned and inserted into the PGL3-control vector by the endonuclease sites XhoI and BamHI to construct PGL3-THBS1-wild type (THBS1-WT) plasmid. The binding site of BZRAP1-AS1 to THBS1 was predicted by the target gene database, while PGL3-THBS1-mutant vector (THBS1-MUT) was constructed by synthesizing the sequence with the binding site mutated. HCC cells at logarithmic growth phase were inoculated into 6-well plates and 24 h later, the cells were separately introduced with oe-BZRAP1-AS1 or sh-BZRAP1-AS1 plus THBS1-WT or THBS1-MUT. Three replicate wells were set for cell co-treatment. After 48 h, the luciferase activity was measured using the dual-luciferase assay kit (RG005, Beyotime Biotechnology Co., Shanghai, China). The luciferase activity was expressed as the ratio of the relative light unit (RLU) of firefly luciferase to the RLU of Renilla luciferase.

### Chromatin immunoprecipitation (ChIP)

The ChIP assay was conducted using the EZ-Magna ChIP TMA kit (Millipore, Billerica, MA, USA). In short, HCC cells at logarithmic growth phase were cross-linked with 1% formaldehyde for 10 min. After being washed twice with pre-cooled PBS, the cells were centrifuged at 715*g* for 5 min and lysed by the cell lysis buffer [150 M NaCl, 50 M Tris (pH = 7.5), 5 μM EDTA, 0.005% NP40, 0.01% Triton X-100] supplemented with the protease inhibitor cocktail. The chromatin was sheared by sonication and the chromatin fragments around 200–1000 bp were obtained. The fragments were centrifuged at 14,000*g* for 10 min at 4 °C. A total of 900 μL ChIP Dilution Buffer and 20 μL 50 × pseudoisocyanine as and 60 μL Protein A Agarose/Salmon Sperm DNA were mixed with 100 μL of the supernatant (DNA fragment) taken from transfected cells at 4 °C for 1 h and allowed to stand for 10 min. After centrifugation, the supernatant was collected with 20 μL serving as the input. The supernatant was incubated with DNMT3b antibody (ab2851, Abcam Inc., Cambridge, UK) at 4 °C overnight. In the negative control group, the DNMT3b antibody was replaced with the rabbit IgG (ab172730, Abcam Inc., Cambridge, UK). The supernatant containing DNA fragments (100 μL) was incubated with 60 μL Protein A Agarose/Salmon Sperm DNA at 4 °C for 2 h. Besides, 20 μL of supernatant was preserved as the input. Subsequently, the mixture was centrifuged and the precipitate was washed with the low salt buffer, the high salt buffer, the LiCl solution, and the trace element solution. The protein-DNA complex was eluted with 250 μL ChIP Wash Buffer and de-crosslinked with 20 μL of 5 M NaCl. DNA was isolated and the content of THTS1 promoter was quantified by fluorescence quantitative PCR. The THBS1 promoter primer sequences used were: F, 5′-ACCGACTTTTCTGAGAAG-3′; R, 5′-GCAACTTTCCAGCTAGAA-3′.

### Xenograft tumor in nude mice

A total of 90 specific pathogen-free female nude mice (aged 4–6 weeks, weighing 20–30 g) were injected with lentiviruses-infected HuH-7 cells. In brief, the cells (6 × 10^6^) were cultured for 24 h and then mixed with Matrigel at a volume ratio of 1:1 before injection. A total of 0.2 mL cell suspension (3 × 10^6^) was injected at the right side axilla of the nude mice. The body weight and tumor volume (V) of mice were measured at an interval of 1 day. The tumor size was calculated using the formula: V (mm^3^) = 1/2L × D^2^, in which L represents the maximum diameter, while D represents the shortest diameter of the tumor. A tumor growth curve was then depicted. These nude mice were euthanized 4 weeks later using excessive CO_2_. The resected tumors were weighted.

### Immunohistochemical staining

The tumor tissues were fixed in 4% paraformaldehyde, embedded in paraffin, and sliced into 5-μm-thick sections. The sections were routinely deparaffinized, followed by antigen retrieval. After being washed with PBS, the sections were blocked by the normal goat serum. Positive HCC sections were taken as the positive control, while IgG instead of the primary antibody was taken as the negative control. The CD31 was detected according to the instructions of Histostain™ SP-9000 immunohistochemical staining kit (Zymed Laboratories, South San Francisco, CA). Primary antibody to CD31 (ab28364, 1:50, Abcam Inc., Cambridge, UK) at 4 °C and goat anti-rabbit secondary antibody (a6721, 1:10,000, Abcam Inc., Cambridge, UK) were used. Subsequently, the sections were added with horseradish-labeled working solution, followed by diaminobenzidine (DAB) development. The sections were then counterstained with hematoxylin for 1 min and mounted with neutral balsam. For the microvessel density (MVD) counting, the most dense area of the neovascularization in the tumor xenografts was chosen as the hotspots for blood vessel counting under a 40-fold low-power microscope and then CD31-stained positive blood vessels in 5 visual fields were counted under a 200-fold high magnification. The positive blood vessels with the interstitial cells stained brown or the nearby cells cluster formed into a vascular shape or a larger blood vessel were recorded. The MVD value was averaged in five visual fields.

### Immunofluorescence staining

Paraffin-embedded tissues were cut into 4-μm-thick sections. The sections were then penetrated and fixed by pre-cooled methanol for 15 min. After blocked with 2% bovine serum albumin and 5% goat serum for 60 min, the sections were incubated with anti-CD31 antibody (ab28364, 1:50, Abcam Inc., Cambridge, UK) at 4 °C overnight and then incubated with Cy3-labeled secondary antibody (ab6939, Abcam Inc., Cambridge, UK) at room temperature for 60 min in the dark. Finally, the tissue sections were stained by 1 mg/mL DAPI. The sections were blocked and stored at 4 °C. Six visual fields were randomly selected to perform the quantitative analysis of CD31 positive cells using Image J software. All images were captured under the LEICA DMRA2 fluorescence microscope (Leica Biosystems, Shanghai, China) equipped with LEICA DC 500 camera.

### Statistical analysis

SPSS 21.0 statistical software (IBM Corp. Armonk, NY, USA) was applied for data analysis. All experiments were repeated three times. The measurement data were expressed as mean ± standard deviation (s.d.). The data normality test was performed using Kolmogorov–Smirnov method. If the data conformed to the normal distribution and homogeneity of variance, unpaired *t* test was used for data analysis between two groups, and one-way analysis of variance (ANOVA) was used for comparison among multiple groups, followed by Tukey’s post hoc tests. The viability at different time points was compared by repeated measures ANOVA. *p* < 0.05 was used as the threshold for statistical significance.

## Results

### BZRAP1-AS1 is highly expressed in HCC with predominant localization in the nucleus

**Fig. 1:**
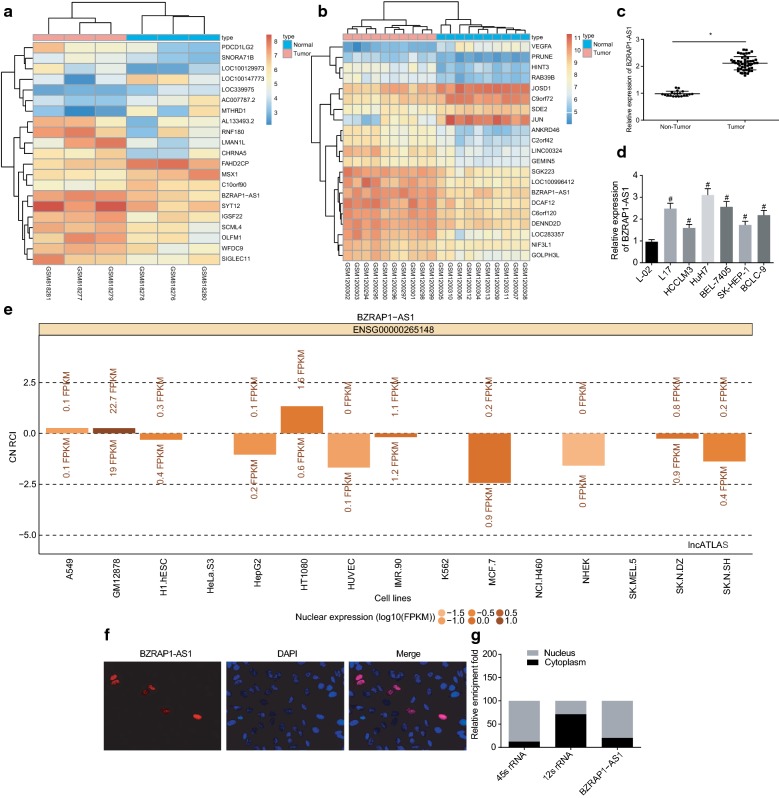
BZRAP1-AS1 is highly expressed in HCC tissues and cells. **a**, **b** Differentially expressed lncRNAs screened from HCC-related microarrays GSE33006 and GSE49515. The abscissa shows the number of sample and the ordinate shows differentially expressed genes. The histogram in the upper right is the color gradation, and each rectangle in the figure corresponds to the expression of one sample. **c** Expression of BZRAP1-AS1 relative to GAPDH in HCC tissues and the adjacent normal tissues. **d** Expression of BZRAP1-AS1 relative to GAPDH in HCC cells and the normal liver cell line. **e** Localization of BZRAP1-AS1 in HCC cells analyzed by lncATLAS online site. **f** Subcellular localization (×200) of BZRAP1-AS1 in HuH-7 cells detected by FISH. **g** Nuclear expression of BZRAP1-AS1 in HuH-7 cells determined by RT-qPCR. *p < 0.05, vs. adjacent normal tissues; ^#^*p* < 0.05 vs. L-02 cell line. The data (mean ± S.D.) obeying the normal distribution variance between two groups were analyzed using unpaired *t* test while those among multiple groups were assessed by one-way ANOVA, followed with Tukey’s post hoc test. The experiment was repeated 3 times independently. *BZRAP1-AS1* BZRAP1-antisense RNA 1, *HCC* hepatocellular carcinoma, *FISH* fluorescence in situ hybridization, *ANOVA* analysis of variance

To uncover lncRNAs that are differentially expressed in HCC, we analyzed the GEO datasets GSE33006 and GSE49515, and identified BZRAP1-AS1 as one of the highly upregulated candidates in HCC. (Figure 1a, b). We confirmed this finding by assessing the expression of BZRAP1-AS1 in HCC tissues, and observed BZRAP1-AS1 expression in HCC tissues was indeed higher than in adjacent normal tissues (*p* < 0.05) (Fig. 1c). Curious whether this aberrant expression of BZRAP1-AS1 is associated with other clinical characteristics of HCC patients, we sought to assess correlations of clinical features of HCC with BZRAP1-AS1 expression and found that BZRAP1-AS1 expression was related to tumor size, microvascular invasion and tumor node metastasis (TNM) stage of HCC patients, but not related to the age, sex, Edmondson’s grade and liver cirrhosis (Table 3). Besides, BZRAP1-AS1 was highly expressed in HCC cell lines compared with the normal liver cell line L-02 (*p* < 0.05) (Fig. 1d). Going forward, we chose the Among the HCC cell lines, HuH-7 presented with the highest expression of BZRAP1-AS1 and was chosen for the subsequent experiments. Moreover, the subcellular localization of BZRAP1-AS1 was predicted by the lncATLAS online website (http://lncatlas.crg.eu/), which revealed that BZRAP1-AS1 might be located in the nucleus of HCC cells (Fig. 1e). Further FISH and RNA analyses verified that BZRAP1-AS1 was mainly detected in the nucleus of HuH-7 cells (Fig. 1f, g). Taken together, our initial set of experiments revealed that BZRAP1-AS1 was localized predominantly in the nucleus and highly expressed in HCC.

**Table 3:** Correlation between the expression of BZRAP1-AS1 and the clinical characteristics of HCC patients

Variable	BZRAP1-AS1 subgroup		
	Low	High	*p* value
All cases	25	24	
Age, years, > 55:< 55	12:13	12:12	0.889
Gender, male/female	19:06	17:07	0.682
Liver cirrhosis, with/without	18:07	14:10	0.315
Tumor size, cm, > 5:< 5	4:21	13:11	0.005*
Edmondson’s grade, I + II:III + IV	16:09	16:08	0.845
Microvascular invasion, present/absent	3:22	9:15	0.038*
TNM stage, I:II:III	18:05:02	9:07:08	0.027*

Data are expressed as ratios. Median expression level was used as the cutoff. Low expression of BZRAP1-AS1 in 23 patients was classified as values below the 50th percentile. High BZRAP1-AS1 expression in 24 patients was classified as values at or above the 50th percentile.*p < 0.05

### Down-regulated BZRAP1-AS1 suppresses HUVEC proliferation and angiogenesis

**Fig. 2:**
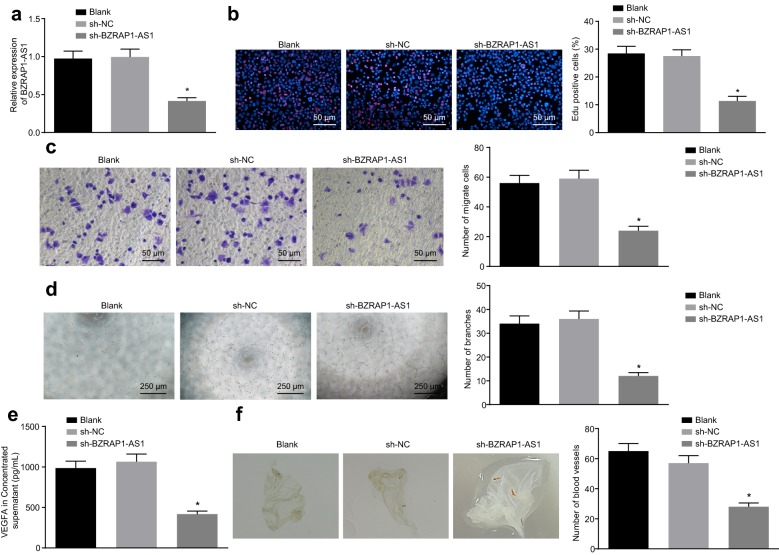
BZRAP1-AS1 silencing suppresses HUVEC proliferation and angiogenesis. **a** The expression of BZRAP1-AS1 in HuH7 cells infected with lentivirus expressing sh-NC or sh-BZRAP1-AS1 determined by RT-qPCR. **b** The effect of BZRAP1-AS1 on the proliferation of HUVECs co-cultured with HuH7 cells revealed by EdU assay (×200). **c** The effect of BZRAP1-AS1 on the migration of HUVECs evaluated by Transwell assay (×40). **d** The effect of BZRAP1-AS1 on angiogenesis of HCC cells assessed by tube formation assay (×50). **e** The level of VEGFA in HuH-7 concentrated supernatant measured by ELISA. **f** The effect of BZRAP1-AS1 on angiogenesis evaluated by CAM assay (×20). *p < 0.05 vs. HuH-7 cells infected with lentivirus expressing sh-NC. The data (mean ± S.D.) obeying the normal distribution and homogeneity of variance among multiple groups were analyzed using one-way ANOVA, followed with Tukey’s post hoc test. The experiment was repeated 3 times independently. *HCC* hepatocellular carcinoma, *BZRAP1-AS1* BZRAP1-antisense RNA 1, *RT-qPCR* reverse transcription quantitative polymerase chain reaction, *EdU* 5-ethynyl-2′-deoxyuridine, *VEGFA* vascular endothelial growth factor, *ELISA* enzyme-linked immunosorbent assay, *CAM* chicken chorioallantoic membrane, *ANOVA* analysis of variance, *NC* negative control, *HUVECs* human umbilical vein endothelial cells

Having discovered a high expression pattern of BZRAP1-AS1 in HCC, we next sought to functionally validate this finding using genetic perturbations. The expression of BZRAP1-AS1 in HuH7 cells was altered by shRNA to evaluate the effect of BZRAP1-AS1 on HUVEC viability and angiogenesis. The expression of BZRAP1-AS1 was then determined in HuH-7 cells with or without treatment of sh-BZRAP1-AS1, which showed that the expression of BZRAP1-AS1 was downregulated in HuH-7 cells transfected with sh-BZRAP1-AS1 (*p* < 0.05) (Fig. 2a).

We then assessed the functional effects of this genetic perturbation on angiogenesis. The ability of HCC cell-induced angiogenesis was evaluated by co-culture of HUVECs with HuH-7 cells. When the level of BZRAP1-AS1 was knocked down, there was a decline in the number of EdU-positive cells (Fig. 2b), migrated cells (Fig. 2c), and tube branches (Fig. 2d), VEGFA content in cell culture medium (Fig. 2e), as well as the number of formed blood vessels (Fig. 2f) (*p* < 0.05). These results collectively supported that silencing of BZRAP1-AS1 inhibited HUVEC proliferation and angiogenesis.

### BZRAP1-AS1 interacts with DNMT3b to reduce THBS1 expression

**Fig. 3:**
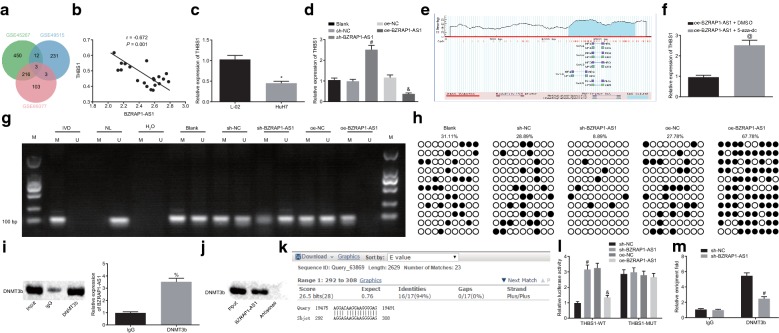
BZRAP1-AS1 inhibits the transcription of THBS1 through mediating the methylation of the THBS1 promoter by interacting with DNMT3b. **a** Venn map of down-regulated genes in HCC in three microarray expression profiles (GSE45267, GSE49515, and GSE89377). **b** Correlation analysis of the expression of BZRAP1-AS1 and THBS1 (GSE49515). **c** mRNA expression of THBS1 in HuH-7 and L-02 cells measured by RT-qPCR. **d** The effect of BZRAP1-AS1 on the transcription of THBS1 assessed by RT-qPCR. **e** Analysis of CpG island enrichment in THBS1 promoter region. **f** mRNA expression of THBS1 when the methylation was inhibited by 5-aza-dc in the presence of BZRAP1-AS1 measured by RT-qPCR. **g** Methylation level of THBS1 promoter measured by the MSP assay. H_2_O was used as double negative control, in vitro methylated DNA (IVD) as positive methylation control, and normal lymphocyte DNA (NL) as unmethylated positive control. U means unmethylation while M means methylation. **h** Methylation level of THBS1 promoter measured by the BSP assay. Black circle stands for methylation site and white circle stands for un-methylated site. **i** Interaction between DNMT3b and BZRAP1-AS1 confirmed by RIP assay. **j** Interaction between BZRAP1-AS1 and DNMT3b verified by RNA pull-down assay. **k** Blast alignment of BZRAP1-AS1 and THBS1 promoter sequences. **l** Interaction between BZRAP1-AS1 and THBS1 promoter region analyzed by dual luciferase reporter gene assay. **m** THTS1 promoter region directly bound to DNMT3b, as detected by the ChIP assay. * *p* < 0.05 vs. L-02 cell line; ^#^*p* < 0.05 vs. cells infected with lentivirus expressing sh-NC; ^&^*p* < 0.05 vs. cells infected with lentivirus expressing oe-NC; ^@^*p* < 0.05 vs. cells infected with lentivirus expressing oe-BZRAP1-AS1 and treated with DMSO; ^%^*p* < 0.05 vs. cells introduced with IgG. The data (mean ± S.D.) obeying the normal distribution and homogeneity of variance between two groups were analyzed using unpaired *t* test while those among multiple groups were assessed by one-way ANOVA, followed with Tukey’s post hoc test. The experiment was repeated 3 times independently. *BZRAP1-AS1* BZRAP1-antisense RNA 1, *THBS1* thrombospondin1, *DNMT3b* DNA methyltransferase 3B, *HCC* hepatocellular carcinoma, *RT-qPCR* reverse transcription quantitative polymerase chain reaction, *MSP* methylation specific PCR, *BSP* bisulfite sequencing PCR, *RIP* RNA immunoprecipitation, *ChIP* chromatin immunoprecipitation, *DMSO* dimethyl sulfoxide, *IgG* immunoglobulin G, *ANOVA* analysis of variance

We then began looking for putative genes under the regulatory control of BZRAP1-AS1 in HCC. The differentially expressed genes were screened from the datasets of GSE45267, GSE49515, and GSE89377 and three intersected genes (FOSB, THBS1, and NAMPT) were identified (Fig. 3a). It was observed that there was a negative correlation between the expression of BZRAP1-AS1 and THBS1 (Fig. 3b). A previous study has validated that THBS1 is an anti-angiogenic factor and could inhibit angiogenesis of tumors [14]. We thus reasoned that it is plausible that the promoting effect of THBS1 on HCC angiogenesis is mediated by BZRAP1-AS1. The results of RT-qPCR showed that the expression of THBS1 was lower in HuH-7 cells than that in L-02 cells (*p* < 0.05) (Fig. 3c), and the expression of THBS1 was up-regulated upon BZRAP1-AS1 knockdown in HuH-7 cells (*p* < 0.05) (Fig. 3d). Next, using the Methprimer website (http://www.urogene.org/cgi-bin/methprimer/methprimer.cgi), we identified a C-phosphate-G (CpG) island in the THTS1 promoter region (Fig. 3e). We next confirmed that the addition of the methyltransferase inhibitor 5-aza-dc attenuated the downregulating effect of BZRAP1-AS1 on THBS1 expression (*p* < 0.05) (Fig. 3f). It was demonstrated in MSP and BSP assays that over-expression of BZRAP1-AS1 promoted the methylation level of THBS1 promoter, while methylation of THBS1 promoter was attenuated by BZRAP1-AS1 silencing (*p* < 0.05) (Fig. 3g, h). Collectively, these results documented that BZRAP1-AS1 regulated the transcriptional expression and methylation of THBS1 in HuH-7 cells, while inhibition of methyltransferase activity could restore the transcription of THBS1.

We next performed RIP, RNA pull-down and ChIP assays to determine the interaction among BZRAP1-AS1, DNMT3b, and THBS1. As the results shown by conducting RIP assay, the presence of BZRAP1-AS1 was detected in RNA co-immunoprecipitated with DNMT3b antibody (*p* < 0.05) (Fig. 3i), and then the presence of the DNMT3b protein was detected using western blot analysis, which further verified that DNMT3b could bind to BZRAP1-AS1 (Fig. 3j). It was revealed that the BZRAP1-AS1 bound to the THBS1 promoter in an RNA–DNA manner (Fig. 3k), which was confirmed by the dual luciferase reporter gene assay. The luciferase activity of THBS1-WT was inhibited after HuH7 cells were infected by lentivirus over-expressing BZRAP1-AS1 (*p* < 0.05), while the luciferase activity of THBS1-MUT remained unaffected by overexpressed BZRAP1-AS1 (*p* > 0.05) (Fig. 3l). Moreover, ChIP data showed that compared to IgG, the content of DNMT3b binding to the THBS1 promoter region was increased (*p* < 0.05) (Fig. 3m), suggesting that DNMT3b was enriched in the THBS1 promoter region, and the enrichment could be attenuated by the knockdown of BZRAP1-AS1.

Taken together, these results demonstrated that BZRAP1-AS1 could interact with DNMT3b and recruited DNMT3b to the promoter region of THBS1, which resulted in hyper-methylation of the CpG site and inhibited transcription of THBS1.

### BZRAP1-AS1 serves as a tumor promoter in HCC via DNMT3b-mediated THBS1

**Fig. 4:**
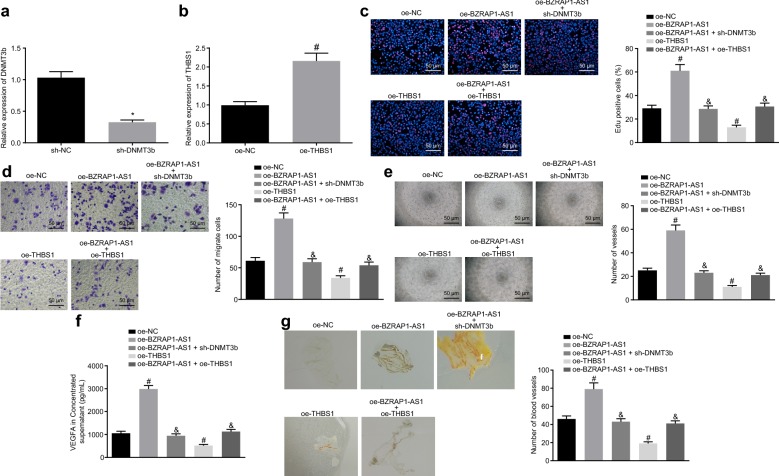
BZRAP1-AS1 regulates the development of HCC by negatively regulating THBS1 via DNMT3b. **a** Transfection efficiency of sh-DNMT3b assessed by RT-qPCR. **b** Transfection efficiency of oe-THBS1 assessed by RT-qPCR. **c** Effects of BZRAP1-AS1, DNMT3b and THBS1 on the proliferation of HUVECs evaluated by EdU assay (×200). **d** Effects of BZRAP1-AS1, DNMT3b and THBS1 on the HUVEC migration evaluated by Transwell assay (×40). **e** Effects of BZRAP1-AS1, DNMT3b and THBS1 on angiogenesis evaluated by tube formation assay (×50). **f** The level of VEGFA in HuH-7 concentrated supernatant analyzed by ELISA. **g** Effects of BZRAP1-AS1, DNMT3b and THBS1 on angiogenesis detected by CAM assay (×20). *p < 0.05 vs. cells infected with lentivirus expressing sh-NC; ^#^*p* < 0.05 vs. cells infected with lentivirus expressing oe-NC; ^&^*p* < 0.05 vs. cells infected with lentivirus expressing oe-BZRAP1-AS1. The data (mean ± S.D.) obeying the normal distribution and homogeneity of variance between two groups were analyzed using unpaired *t* test while those among multiple groups were assessed by one-way ANOVA, followed with Tukey’s post hoc test. The experiment was repeated 3 times independently. *BZRAP1-AS1* BZRAP1-antisense RNA 1, *DNMT* DNA methyltransferase, *HCC* hepatocellular carcinoma, *THBS1* thrombospondin1, *RT-qPCR* reverse transcription quantitative polymerase chain reaction, *DNMT3b* DNA methyltransferase 3B, *EdU* 5-ethynyl-2′-deoxyuridine, *VEGFA* vascular endothelial growth factor, *ELISA* enzyme-linked immunosorbent assay, *CAM* chicken chorioallantoic membrane, *ANOVA* analysis of variance, *NC* negative control

Given that knockdown of BZRAP1-AS1 in HuH-7 cells could suppress the proliferation, migration and angiogenesis of HUVEC cells, we reasoned that BZRAP1-AS1 could lead to hyper-methylation at the CpG site and gene silencing by recruiting DNMT3b to the THBS1 promoter region. Therefore, we hypothesized that BZRAP1-AS1 played a role in HCC by regulating THBS1. The mRNA level of DNMT3b in HuH-7 cells was decreased by infection of lentivirus expressing sh-DNMT3b (*p* < 0.05) (Fig. 4a), while that of THBS1 in HuH-7 cells infected with lentivirus expressing oe-THBS1 was increased (*p* < 0.05) (Fig. 4b). We then observed that upon down-regulating the level of DNMT3b or up-regulating the expression of THBS1, the number of EdU-positive cells, migrated cells and branches, VEGFA content in cell culture medium as well as the number of formed blood vessels was decreased in HuH-7 cells overexpressing BZRAP1-AS1 (*p* < 0.05), respectively revealed by EdU assay (Fig. 4c), Transwell assay (Fig. 4d), tube formation assay (Fig. 4e), ELISA (Fig. 4f) and CAM assay (Fig. 4g). Collectively, these results suggested that the promoting role of BZRAP1-AS1 in the proliferation, migration and angiogenesis of HUVEC cells could be inhibited by silencing of DNMT3b or restoration of THBS1, and the function of BZRAP1-AS1 on the progression of HuH-7 cells was achieved by recruiting DNMT to mediate the THBS1 methylation.

### BZRAP1-AS1 promotes the angiogenesis of HCC in vivo via mediating THBS1

**Fig. 5:**
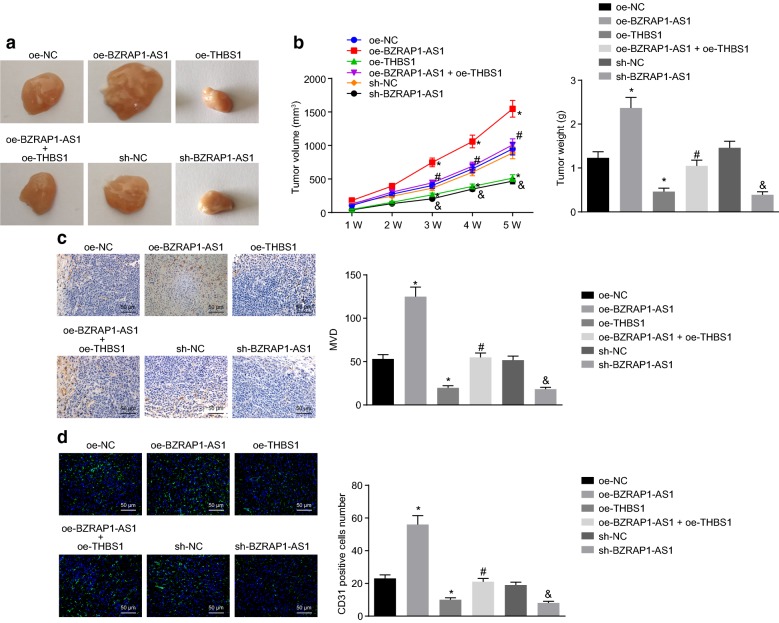
BZRAP1-AS1 silencing represses the angiogenesis of HCC in vivo. **a** Xenograft tumor growth curve. **b** Tumor volume and weight at the 35th day after injection. **c** MVD and the level of CD31 determined by immunohistochemical staining (×200). **d** Tumor angiogenesis in vivo determined by immunofluorescence staining (×500). *p < 0.05 vs. mice injected with oe-NC-treated cells; ^#^*p* < 0.05 vs. mice injected with oe-THBS1-treated cells; ^&^*p* < 0.05 vs. mice injected with sh-NC-treated cells. The data (mean ± S.D.) obeying the normal distribution homogeneity of variance among multiple groups were assessed by one-way ANOVA and repeated measures ANOVA was performed for proliferation ability at different time points followed with Tukey’s post hoc test. The experiment was repeated 3 times independently. n = 15. *HCC* hepatocellular carcinoma, *MVD* microvessel density, *ANOVA* analysis of variance

To further investigate the effect of BZRAP1-AS1 on the development of HCC in vivo, BABL/C nude mice were used to simulate the in vivo environment in the present study. The results demonstrated that the tumor volume and weight of mice were decreased by over-expression of THBS1 or silencing of BZRAP1-AS1 (*p* < 0.05). Besides, the tumor volume and weight of mice inhibited by over-expression of THBS1 could be eliminated by over-expression of BZRAP1-AS1 (*p* < 0.05) (Fig. 5a, b). These results therefore clarified that BZRAP1-AS1 could promote the development of HCC in vivo through increasing THBS1.

Next, we performed immunohistochemical staining and found that the MVD value was decreased in response to over-expression of THBS1 or silencing of BZRAP1-AS1 (*p* < 0.05). Additionally, over-expression of BZRAP1-AS1 attenuated the suppressive effect of THBS1 over-expression on MVD value (*p* < 0.05) (Fig. 5c). The expression of CD31 in tumors was determined by immunofluorescence staining, and the results clarified that CD31 was reduced when THBS1 was up-regulated or BZRAP1-AS1 was silenced (*p* < 0.05). Moreover, over-expressed BZRAP1-AS1 increased the level of CD31 and attenuated the inhibitory effect of THBS1 on the expression of CD31 (*p* < 0.05) (Fig. 5d). Taken together, these data indicate that silencing of BZRAP1-AS1 can suppress tumor angiogenesis in vivo through up-regulation of THBS1.

## Discussion

HCC is a leading cause of cancer-related mortality worldwide and patients with HCC are commonly diagnosed at an advanced stage, for which highly effective therapies are limited [17]. Accumulating studies have highlighted the implication of lncRNAs in the tumor angiogenesis and their potential as biomarkers or therapeutic targets in multiple cancers [18]. This study focused on the role of lncRNA BZRAP1-AS1 in the tumor angiogenesis of HCC. Our results suggested that BZRAP1-AS1 was highly expressed in HCC and inhibited the transcription of THBS1 through recruiting DNMT3b on its promoter region. Moreover, our study provides evidence for the pro-angiogenic effects of BZRAP1-AS1 on HCC.

The first observation of our study was that BZRAP1-AS1 was highly expressed in both HCC tissues and cells. Partially consistent with our observation, BZRAP1-AS1 was found to be up-regulated in prostate cancer samples and may be a novel biomarker associated with prostate cancer [11]. LncRNAs have been extensively reported to play an important role in angiogenesis; for instance, lncRNA MVIH has been shown to activate angiogenesis [13]. The survival of patients with HCC remains unfavorable, which is largely due to active angiogenesis [19]. Another oncogenic lncRNA UBE2CP3 has been found to induce the secretion of VEGFA and accelerate the angiogenesis, in addition to the enhanced tube formation capabilities of HUVECs in HCC [20]. In our study, BZRAP1-AS1 was detected to enhance angiogenesis as evidenced by an increased VEGFA expression and accelerated tube formation in HCC cells following overexpressed BZRAP1-AS1 treatment. In addition, loss of BZRAP1-AS1 could restrain viability of HUVECs in vitro. Similarly, a lncRNA associated to HCC CRNDE has the capacity to potentiate HCC cell proliferation, migration and invasion in vitro, which can be abolished by ablation of CRNDE [21]. Furthermore, in vivo experimental results in the current study further validated that shRNA-mediated silencing of BZRAP1-AS1 restrained tumor angiogenesis in HCC.

Additionally, BZRAP1-AS1 was observed to recruit DNMT3b on the promoter region of THBS1, by which BZRAP1-AS1 can induce THBS1 DNA methylation, thereby repressing the transcription of THBS1. DNA methylation that generally exists at palindromic CpG sites in mammals can mediate chromatin structure and function, and is crucially involved in epigenetic network [22]. DNMTs, including DNMT1 and DNMT3A, have been shown to induce the methylation of both unmethylated and hemimethylated DNA in cells [23, 24]. The recruitment of another DNMT member, DNMT3b is mediated by a variety of mechanisms such as chromatin modifications, transcription levels, non-coding RNAs, and DNA-binding factors [25]. DNA methylation abnormalities are a common mark of human diseases involving chromosomal and genomic instabilities, such as inherited diseases and cancers [26]. Consistent with our results, inhibition of THBS1 induced by DNA hypermethylation shares an association with tumor progression in laryngeal squamous cell carcinoma [27]. LncRNA LUCAT1 affects the stability of DNMT1 and decreases the expression of tumor suppressor genes in a way of DNA methylation, thereby inducing tumorigenesis in esophageal squamous cell carcinoma [28]. A number of lncRNAs have been demonstrated to act as manipulators for DNA methylation or scaffolds for histone modification to affect key signaling pathways in hepatocarcinogenesis [29].

Furthermore, inhibition of BZRAP1-AS1 impeded angiogenesis of HUVECs through up-regulation of THBS1 via recruiting DNMT3b. Lower THBS1 expression is related to advanced grade of liver and lymph node metastases, coupled with worse overall survival [30]. THBS1 underlies the inhibitory role of BMP4 in tumor angiogenesis, which is mainly achieved by a network that VEGF expression is reduced by BMP4 in a THBS1-dependent manner in the tumor microenvironment [31]. Histone deacetylase-2 (HDAC2) epigenetically represses the expression of THBS1, consequently inducing angiogenesis in prostate cancer [32]. Previous evidence has also shown that THBS1 is an anti-angiogenic factor, which can inhibit angiogenesis of tumors [15]. Accumulating evidence indicates that lncRNAs act as molecular switches in cellular biological functions and in the reprogramming of cell states via the alteration of gene expression patterns [13]. Therefore, in summary, BZRAP1-AS1 may affect angiogenesis of HCC by regulating the expression of THBS1 both in vitro and in vivo.

## Conclusion

**Fig. 6:**
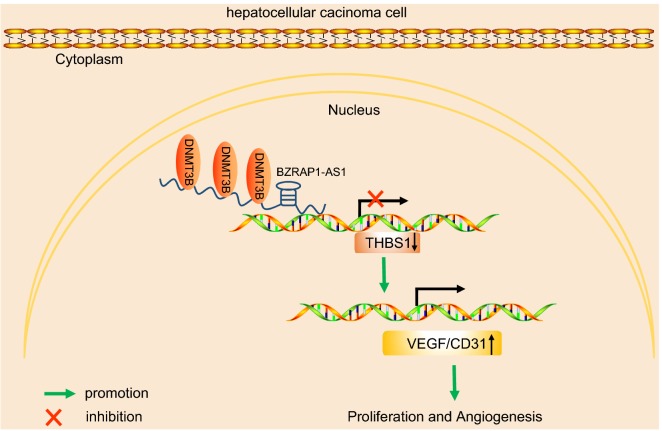
The mechanism map depicting that BZRAP1-AS1 promotes the progression of HCC through down-regulation of THBS1 via recruiting DNMT3b. BZRAP1-AS1, highly expressed in HCC, binds to the promoter of THBS1, recruits the DNMT3b, and enhances the methylation of THBS1 promoter, thus inhibiting the transcription of THBS1. THBS1 depletion promotes angiogenesis via increasing VEGFA and CD31 expression. *HCC* hepatocellular carcinoma, *BZRAP1-AS1* BZRAP1-antisense RNA 1, *THBS1* thrombospondin1

In conclusion, the present study demonstrates that BZRAP1-AS1 silencing impedes tumor angiogenesis through increase of THBS1 (Fig. 6). BZRAP1-AS1 can be a potential therapeutic target to restrain tumor growth in HCC. Although the findings may lack clinical evidence, our study still have implications for anti-angiogenic and anti-tumor treatment strategies.

## Data Availability

The datasets generated/analysed during the current study are available

## Abbreviations

HCC: hepatocellular carcinoma
lncRNAs: long non-coding RNAs
THBS1: thrombospondin-1
MSP: methylation-specific PCR
BSP: bisulfite sequencing PCR
BZRAP1-AS1: benzodiazapine receptor associated protein 1 antisense RNA 1
HBV: hepatitis B virus
HCV: hepatitis C virus
ncRNAs: non-coding RNAs
FBS: fetal bovine serum
GFP: green fluorescent protein
DMEM: Dulbecco’s modified eagle’s medium
RPMI: Roswell Park Memorial Institute
oe: overexpressed
sh: short hairpin
GAPDH: glyceraldehyde-3-phosphate dehydrogenase
PBS: phosphate buffered saline
TBST: Tris-buffered saline Tween-20
HUVECs: human umbilical vein endothelial cells
RT-qPCR: reverse transcription quantitative polymerase chain reaction
cDNA: complementary DNA
FISH: fluorescence in situ hybridization
DAPI: 4′-6-diamidino-2-phenylindole
EDTA: ethylenediamine tetraacetic acid
EdU: 5-ethynyl-2′-deoxyuridine
ELISA: enzyme-linked immunosorbent assay
CAM: chicken chorioallantoic membrane
RIP: RNA binding protein immunoprecipitation
IgG: immunoglobulin G
ChIP: chromatin immunoprecipitation
OD: optical density
CG: cytosine guanine
RLU: relative light unit
WT: wild type
MUT: mutant
DAB: diaminobenzidine
MVD: microvessel density
s.d.: standard deviation
ANOVA: analysis of variance
HOTAIR: HOX transcript antisense intergenic RNA
TNM: tumor node metastasis
CpG: C-phosphate-G
